# Periprosthetic Femur Fractures Managed by Revision Arthroplasty

**DOI:** 10.7759/cureus.87204

**Published:** 2025-07-02

**Authors:** Uday Mahajan, Meraj Akhtar, Kashif Memon

**Affiliations:** 1 Trauma and Orthopaedics, Queen Elizabeth Hospital Birmingham, Birmingham, GBR; 2 Trauma and Orthopedics, Lincoln County Hospital, Lincoln, GBR

**Keywords:** femoral replacement prosthesis, hip revision arthroplasty, interprosthetic fracture, periprosthetic femoral fracture (pff), surgical outcomes

## Abstract

Background

Periprosthetic femoral fractures (PFFs) are a growing indication for revision total hip arthroplasty (THA), particularly in elderly patients with complex comorbidities. Surgical management is challenging, with significant morbidity and mortality. This study evaluates outcomes following revision arthroplasty for PFFs at a tertiary trauma centre in the United Kingdom .

Methods

A retrospective review was conducted of 24 patients who underwent revision arthroplasty for PFFs between February 2021 and January 2022. Data collected included patient demographics, fracture classification, implant history, surgical details, and complications. Follow-up data were extracted from electronic health records up to February 2025. The primary outcome was the need for further revision; secondary outcomes included mortality and postoperative complications.

Results

The cohort had a mean age of 78.3 years (IQR: 16), with most injuries resulting from low-energy trauma. Vancouver B-type fractures were the most common, and three patients sustained interprosthetic fractures. Surgical strategies included modular fluted stems (n=13), femoral replacement prostheses (n=7), and cemented stems (n=4). Complications included one infection managed with suppressive antibiotics, two recurrent dislocations, and two cases of leg length discrepancy. One patient required further revision for instability. Twelve patients died during follow-up: two within 30 days, three within one year, and seven after one year. Thirteen patients were followed remotely due to frailty or relocation.

Conclusion

Revision arthroplasty for PFFs involves technically demanding procedures with significant risks. Despite the complexity, outcomes were in line with published data. Multidisciplinary care, timely surgical intervention, and individualised implant selection remain critical to optimise outcomes in this high-risk population.

## Introduction

Periprosthetic femoral fractures (PFFs) are a growing clinical challenge, now recognised as the most common indication for revision total hip arthroplasty (THA) [1]. According to the National Joint Registry, revision procedures for PFFs have nearly doubled over the past decade [2]. With the ongoing rise in both primary and revision THA procedures, the incidence of PFFs is projected to increase by approximately 4.6% per decade over the next 30 years [3,4]. These epidemiological trends underscore the need for robust strategies to manage PFFs effectively, particularly in high-risk elderly patients with compromised bone quality [5].

Recent literature highlights the substantial morbidity and mortality associated with periprosthetic femoral fractures (PFFs), with one-year mortality rates reported as high as 13-21%, mirroring those of native geriatric hip fractures [6-8]. A multicentre UK cohort study further confirmed that the 12-month mortality following PFFs was 21%, with the risk exacerbated in patients admitted from care homes or undergoing nonoperative management [8]. Key prognostic factors influencing mortality include advanced age, multiple comorbidities, poor preoperative functional status, and surgical delays [6,7]. Additionally, the clinical management of PFFs remains complex, frequently challenged by prosthetic loosening, re-fracture, and periprosthetic joint infection. These complications significantly impair outcomes and increase the likelihood of re-revision procedures, emphasizing the importance of individualized surgical planning and timely multidisciplinary intervention [7,9].

Treatment approaches depend on fracture classification and implant stability, with revision arthroplasty generally favoured for fractures involving a loose prosthesis or failed fixation [10]. However, revision arthroplasty carries a notable risk of complications, including dislocation and deep infection, often managed with long-term antibiotic suppression, underscoring the importance of meticulous preoperative planning and appropriate patient selection [11,12]. This study aims to evaluate the clinical outcomes of patients undergoing revision arthroplasty for periprosthetic femoral fractures at a tertiary trauma centre in the UK, with a specific focus on mortality rates, postoperative complications, surgical timing, and the need for further revision procedures.

## Materials and methods

This retrospective study reviewed 24 consecutive patients who underwent revision arthroplasty for periprosthetic femoral fractures (PFFs) at a UK major trauma centre between February 2021 and January 2022. All procedures were performed by fellowship-trained orthopaedic consultants with experience in revision hip surgery. Patient data were obtained from operative logs and electronic health records and included demographic characteristics, fracture classification using the Vancouver system, type of primary hip arthroplasty, time interval between index surgery and fracture, time to revision surgery, and prosthesis used for reconstruction. Additional details captured included perioperative complications, mortality, and need for re-revision. All data were anonymised, and medical records were reviewed in a de-identified format to ensure confidentiality and compliance with institutional governance standards.

Inclusion criteria were adults undergoing revision arthroplasty for PFFs involving a loose or failed femoral stem. Patients managed with open reduction and internal fixation (ORIF), those undergoing revision for periprosthetic joint infection, and fractures associated with malignancy were excluded. The decision to proceed with revision arthroplasty rather than internal fixation was made based on preoperative imaging and intraoperative assessment of implant stability, the presence of a loose femoral stem, the extent and location of the fracture, and the quality of remaining bone stock, ensuring optimal implant selection and construct stability.

Postoperative follow-up included clinic visits and radiographs, where available. For patients unable to attend in-person follow-up due to frailty or relocation, outcomes were assessed via review of electronic medical records. Remote follow-up included documentation of complications, hospital readmissions, further surgery, and mortality. Electronic records were reviewed up to February 2025 to maximise completeness. Continuous variables are reported as means and interquartile ranges (IQRs), and categorical data as frequencies and percentages.

## Results

The study cohort included 24 patients (12 male, 12 female), with a mean age of 78.3 years (IQR: 16). The predominant mechanism of injury was low-energy trauma, with 21 patients sustaining a fall from standing height. The other mechanisms of injury included one low-speed road traffic collision and two iatrogenic fractures sustained intraoperatively during closed reduction of dislocated prosthetic hips. Fractures were classified using the Vancouver system: two type A, 15 type B (predominantly B2), and three type C fractures. Three additional cases involved fractures around hip resurfacing implants (Birmingham Hip Resurfacing, BHR). Interestingly, three out of the 24 patients sustained interprosthetic fractures between a femoral stem and an ipsilateral total knee replacement (TKR). 

Regarding prior implants, 10 patients had cemented THA, eight had uncemented THA, three had undergone hip resurfacing, and three had hemiarthroplasties. Fractures following cemented THA occurred at a mean of 8.8 years post-surgery, compared to 6.2 years for uncemented THA. Hemiarthroplasty-associated fractures occurred sooner (mean: 1.1 years), while those following hip resurfacing occurred later (mean: 15 years). The mean time to surgical intervention was five days (range: 1-14 days). The mean interval between the primary arthroplasty and the occurrence of PFF was 6.8 years (IQR: 8), with individual cases ranging from two days to 24 years. These cases highlight the complexity of revision procedures, which often require advanced implants such as modular fluted tapered stems of varying lengths for diaphyseal fixation in 13 cases, polished cemented stems in selected cases in three cases, and femoral replacement prostheses for extensive bone loss in seven patients. The results are summarised in Table 1.

**Table 1:** Summary of results THA - total hip arthroplasty, BHR - Birmingham hip replacement (resurfacing), ITU - Intensive therapy unit.

Variable	Value
Mean Age	78.3 years (IQR: 16)
Gender (Male:Female)	12:12
Vancouver Classification	B1: 6, B2: 7, B3: 2, C: 4, A: 2, BHR: 3
Cemented vs. Uncemented THA	Cemented: 7, Uncemented: 11
Hip Resurfacing Cases	3
Hemiarthroplasty Cases	5
Mean Delay to Surgery	6.2 days (range: 1–14)
Post-op Ward vs. ITU Admission	Ward: 20, ITU: 4
Mortality at 30 days	2 patients (8.3%)
Mortality at 1 year	3 patients (12.5%)

Documented complications included one case of deep infection in a patient unfit for reoperation, managed with long-term suppressive antibiotics, two cases of leg length discrepancy not requiring further intervention beyond a shoe raise, and two patients with recurrent dislocations. One of these patients underwent a revision procedure involving a dual mobility cup, while the other was deemed unfit for further surgery and managed non-operatively. A total of 12 patients died during the follow-up period: two within the first 30 days postoperatively, three within the first year, and seven at intervals beyond one year. All deaths were due to unrelated medical causes or general medical decline, reflecting the frailty and comorbidity burden in this cohort. Follow-up data were variable, with 13 patients not having in-person clinic follow-up; instead, their postoperative course was monitored remotely through electronic health records due to health decline or relocation. Electronic records were reviewed through February 2025 to ensure comprehensive outcome capture.

## Discussion

The demographic profile of our cohort, with a mean age of 78.3 years and an even gender distribution, mirrors the population most vulnerable to periprosthetic femoral fractures (PFFs), as shown in prior epidemiological studies [13]. The predominance of low-energy mechanisms, primarily falls from standing height, underscores the frailty and limited physiological reserve of these patients [14]. Our observed 30-day and one-year mortality rates (8.3% and 12.5%, respectively) are consistent with those reported in large meta-analyses, by Lamb et al. including one of 4,841 patients showing pooled 30-day, 90-day, and one-year mortality rates of 3.3%, 4.8%, and 13.4%, respectively [15]. These outcomes are further corroborated by the COMPOSE study, which reported a 12-month mortality of 21% among elderly patients with PFFs in the UK [8]. Additional multicenter data by Moreta et al. [16] confirmed similarly high mortality rates, while Boylan et al. [17] demonstrated that mortality following periprosthetic proximal femoral fractures may even surpass that of native femoral neck fractures in older adults. Furthermore, El Khassawna et al. emphasized the importance of comorbidity indices, such as the Charlson Comorbidity Index, in predicting postoperative outcomes in these patients [18]. These findings collectively underscore the importance of prompt surgical intervention, meticulous risk assessment, and coordinated multidisciplinary care in improving outcomes in this high-risk demographic.

Fracture morphology in our cohort was dominated by Vancouver B-type injuries, with B2 fractures constituting the majority. This pattern aligns with large registry and institutional series identifying B2 fractures as the most frequent subtype necessitating revision arthroplasty due to femoral component loosening [9]. Notably, we identified three interprosthetic fractures-those occurring between a hip stem and an ipsilateral total knee replacement-an increasingly recognized and surgically challenging entity. These fractures present unique difficulties due to limited diaphyseal bone stock, altered biomechanical loading, and stress risers created by adjacent implants [19]. The inclusion of patients with varied prior arthroplasties, including hip resurfacing, hemiarthroplasty, and both cemented and uncemented THAs, permitted a comparative view of temporal fracture patterns. Fractures following hip resurfacing were notably delayed, reflecting the initial durability of these constructs before mechanical fatigue or bone remodeling contributes to late failure [20]. In contrast, fractures occurring after hemiarthroplasty were clustered early postoperatively, likely reflecting patient frailty, reduced bone mineral density, and altered load transfer due to the monoblock nature of these prostheses [21].

The range of implants used in this series highlights the complexity and heterogeneity of revision strategies required for PFFs. Modular fluted tapered stems were most employed, offering flexibility in achieving diaphyseal fixation and restoration of length and offset. These implants are considered a standard solution in revision settings, particularly for B2 and B3 fractures, and have been shown to reduce the risk of aseptic loosening [22]. Femoral replacement prostheses were used in nearly one-third of cases due to extensive bone loss, reflecting the severity of osseous compromise in select patients. The use of cemented polished stems was limited to cases where bone quality and geometry permitted reliable fixation. These decisions reinforce the importance of tailoring surgical planning to individual fracture morphology, implant stability, and host factors, in line with best-practice guidelines.

Despite the technical complexity of revision arthroplasty in this cohort, the overall complication rate was low. Only one patient developed a deep infection, which was managed non-operatively with long-term suppressive antibiotics due to poor surgical fitness. Two patients experienced recurrent dislocations, with one successfully treated using a dual mobility cup and the other managed conservatively. Dislocations were observed in the patient who underwent proximal femoral replacement. This is consistent with published literature, which reports higher dislocation rates following proximal or total femoral replacement due to the loss of soft tissue attachments and compromised abductor function [23]. A leg length discrepancy was noted in two cases but did not necessitate further surgical intervention beyond orthotic adjustment. These findings are consistent with other series reporting complication rates of 10-20% following revision for PFF, though variability in definitions and follow-up intervals complicates comparison [24]. Notably, the high proportion of patients requiring remote follow-up due to frailty or relocation highlights the importance of comprehensive electronic health record review in retrospective analyses, while also limiting functional outcome assessment.

This study has several limitations. Its retrospective design and relatively small sample size limit the generalisability of findings. Functional outcome measures, such as mobility or quality-of-life scores, were not consistently available due to variability in patient frailty and follow-up arrangements. A substantial proportion of patients were monitored remotely using electronic health records rather than in-person review, which, although valuable for capturing mortality and complications, limited detailed clinical assessment. Furthermore, the heterogeneity in implant types and fracture patterns prevented direct comparison between treatment subgroups. Despite these limitations, the study provides meaningful insight into the real-world complexity of managing periprosthetic femoral fractures with revision arthroplasty in a high-risk population.

## Conclusions

Periprosthetic femoral fractures remain a complex and high-risk complication of hip arthroplasty, particularly in older patients with multiple comorbidities. This study reinforces the challenges associated with managing these injuries, including surgical complexity, variable implant requirements, and significant mortality risk. Despite a heterogeneous cohort and reliance on electronic follow-up in many cases, outcomes were broadly consistent with existing literature. These findings support the role of tailored surgical strategies and timely multidisciplinary care in optimising outcomes following revision arthroplasty for PFFs.
